# Scapular stabilization exercise based on the type of scapular dyskinesis versus traditional rehabilitation training in the treatment of periarthritis of the shoulder: study protocol for a randomized controlled trial

**DOI:** 10.1186/s13063-021-05654-2

**Published:** 2021-10-18

**Authors:** Lan Tang, Kang Chen, Yuhui Ma, Lihua Huang, Juan Liang, Yanhong Ma

**Affiliations:** Department of Rehabilitation Medicine, Shanghai Jiao Tong University Affiliated Sixth People’s Hospital, 600 Yishan Rd, Shanghai, 200233 China

**Keywords:** Scapular stabilization exercise, Scapular dyskinesis, Periarthritis of the shoulder, Muscle imbalance

## Abstract

**Background:**

Periarthritis of the shoulder is a common disease leading to dysfunction of the shoulder joint and have a significant impact on patients’ daily life. Evidence shows that there is a close relationship between scapular dyskinesis (SD) and shoulder diseases. Scapular stabilization exercise has been proved to be efficacious in relieving pain and improving function. However, there is no targeted exercise based on the type of scapular dyskinesis. This study will investigate the potential of scapular stabilization exercise based on the type of scapular dyskinesis in treating periarthritis of the shoulder.

**Methods:**

This study is a prospective, randomized controlled, parallel-group trial, intending to recruit 90 patients diagnosed with periarthritis of the shoulder. Patients will receive scapular stabilization exercise training based on the type of scapular dyskinesis or receive traditional rehabilitation training conducted for 30 min, once a day, for 6 weeks. The primary outcome is Constant-Murley score (CMS), and other outcomes include pain degree, range of motion (ROM), type of scapular dyskinesis, scapula position, and patients’ satisfaction with shoulder function. Assessments will be performed at baseline, 2-, 4- and 6-week treatment, and at the 6-week follow-up after the end of treatment.

**Discussion:**

This study will be the first study to investigate the clinical efficacy of scapular stabilization exercise based on the type of scapular dyskinesis in patients with periarthritis of the shoulder. The results may provide evidence of the effect of targeted scapular stabilization exercise in improving shoulder function and correcting scapular dyskinesis, and provide valuable information for future research.

**Trial registration:**

This study had been registered in the Chinese Clinical Trials Registry. Registration number: ChiCTR2100044332 at March 14, 2021.

## Background

Generalized periarthritis of the shoulder is a sterile specific inflammatory reaction caused by long-term chronic injury or degeneration of the joint capsule around the shoulder joint and surrounding tendons, muscles, ligaments and other soft tissues [1]. It includes rotator cuff injuries, biceps tendonitis, supraspinatus tendonitis, shoulder impingement syndrome, shoulder instability, calcified tendonitis, acromioclavicular arthritis, and sternoclavicular arthritis. The annual incidence of PA is between 3 and 5% in the general population and it usually develops between the ages of 40 to 70 years. It is more common in women than men [2]. Patients often suffer from recurrent pain and limited shoulder movement, which affects daily life and work and brings economic burdens on individuals and societies [3]. Current conventional therapies include non-steroidal medications, physical agent therapy, and joint cavity injections, while the efficacy remains unsatisfactory [4]. In addition, joint cavity injections may lead to adverse events such as infectious arthritis [5].

On the other hand, the scapula plays an important role in maintaining complex shoulder kinematics [6]. Altered scapular motion and position have been termed scapular dyskinesis (SD) [7]. Kibler et al. [8] classified SD into 4 types. Type-1 represents the dorsal prominence of the inferior angle of the scapula. Type-2 represents the dorsal prominence of the entire medial border. Type-3 represents the elevated superior border of the scapula, and the scapula could also be anteriorly displaced from the posterior thorax. Type-4 represents the symmetry of bilateral scapula. According to data reported in the literature, SD may be important risk factors for periarthritis of the shoulder [9–11]. The scapular muscle group, consisting of the trapezius, serratus anterior (SA), pectoralis minor (PM), levator scapulae (LS), rhomboid muscle (RM), and teres major (TM), is mainly responsible for scapular movement and dynamic stabilization of the scapula. An optimal interaction between these muscles is needed to provide stability and mobility of the scapula both at rest and during shoulder movements [11]. Previous studies have reported excessive activation of upper trapezius (UT), PM, LS, TM, and decreased activity of middle/lower trapezius (MT/LT), SA, RM in subjects with SD, and different types of SD have different manifestations of muscle imbalance [12–17]. These altered muscle activation patterns are associated with altered scapular kinematics, including reduced scapular upward rotation, external rotation and posterior tilt [12]. Overall, SD is closely related to muscle imbalance and is usually associated with shoulder pathology [18]. However, it is unclear if SD is the cause or the result of shoulder pathology.

The significance of scapular stabilization exercise has drawn considerable attention from research scientists and clinicians, and some studies have not revealed that scapular stabilization exercise is effective in improving shoulder function [19–22] (Table 1). The purpose of scapular stabilization exercise is to restore the position, direction, muscle movement control, and movement pattern of the scapula to stabilize the scapula and improve shoulder joint function. Current stabilization exercise focuses on overall stretching and strengthening of periscapular muscles to improve muscle imbalance and muscle activation. However, different types of SD have different manifestations of muscle imbalance. Thus, there is a lack of targeted scapular stabilization exercise based on the type of SD.

**Table 1:** Characteristics of studies about the efficacy of scapular stabilization exercise

Study	Participants	Interventions (scapular stabilization exercise)	Outcome measures	Conclusions
Struyf et al. (2013) [20]	22 patients with shoulder impingement syndrome	Manual mobilization of scapula; stretching exercises for levator scapulae, rhomboid and pectoralis minor; scapular motor control training (including training of the trapezius and SA muscles).	Shoulder disability questionnaire, verbal numerical rating scale, visual analogue scale, visual observation for tilting & winging, forward head posture, pectoralis minor muscle length, scapular upward rotation.	Scapular stabilization exercise is effective in reducing pain and disability for patients with shoulder impingement syndrome.
Baskurt et al. (2011) [19]	40 shoulder impingement patients	Scapular PNF exercise, scapular clock exercise, standing weight shift, double arm balancing, scapular depression, wall push-up, wall slide exercise.	Visual analogue scale, shoulder range of motion, trapezius and serratus anterior muscle strength, joint position test, Western Ontario Rotator cuff index.	Scapular stabilization exercises are superior to conventional program.
Turgut et al. (2017) [21]	30 subacromial impingement syndrome patients	Wall slides with squat, wall push-ups plus ipsilateral leg extension, lawnmower with diagonal squat, resisted scapular retraction with contralateral 1-leg squat, and robbery with squat.	Three-dimensional kinematics, the Turkish version of the Shoulder Pain and Disability Index (SPADI).	Scapular stabilization exercise therapy is an effective tool for controlling pain and improving disability status.
Hotta et al. (2018) [22]	50 patients with shoulder impingement	The neuromuscular exercises were towel slide, PNF scapular, inferior glide, and scapular clock. The strengthening exercises were diagonal D1, push-up plus, full can, prone horizontal abduction with external rotation from 90° to 135°, side-lying external rotation with abduction at 0°, diagonal D2 eccentric, scapular punch, and horizontal rowing.	Resting position and scapular kinematics, muscular strength, numeric pain rating scale, Shoulder Pain and Disability Index (SPADI).	Motor control and muscular strengthening training improve function among subjects with shoulder impingement syndrome.

In the present study design, we developed a randomized controlled trial to evaluate the effect of scapular stabilization exercise based on the type of SD in patients with periarthritis of the shoulder. We hypothesized that scapular stabilization exercise based on the type of SD would improve shoulder function and correct SD better than traditional rehabilitation training.

## Methods

### Study design

**Fig. 1:**
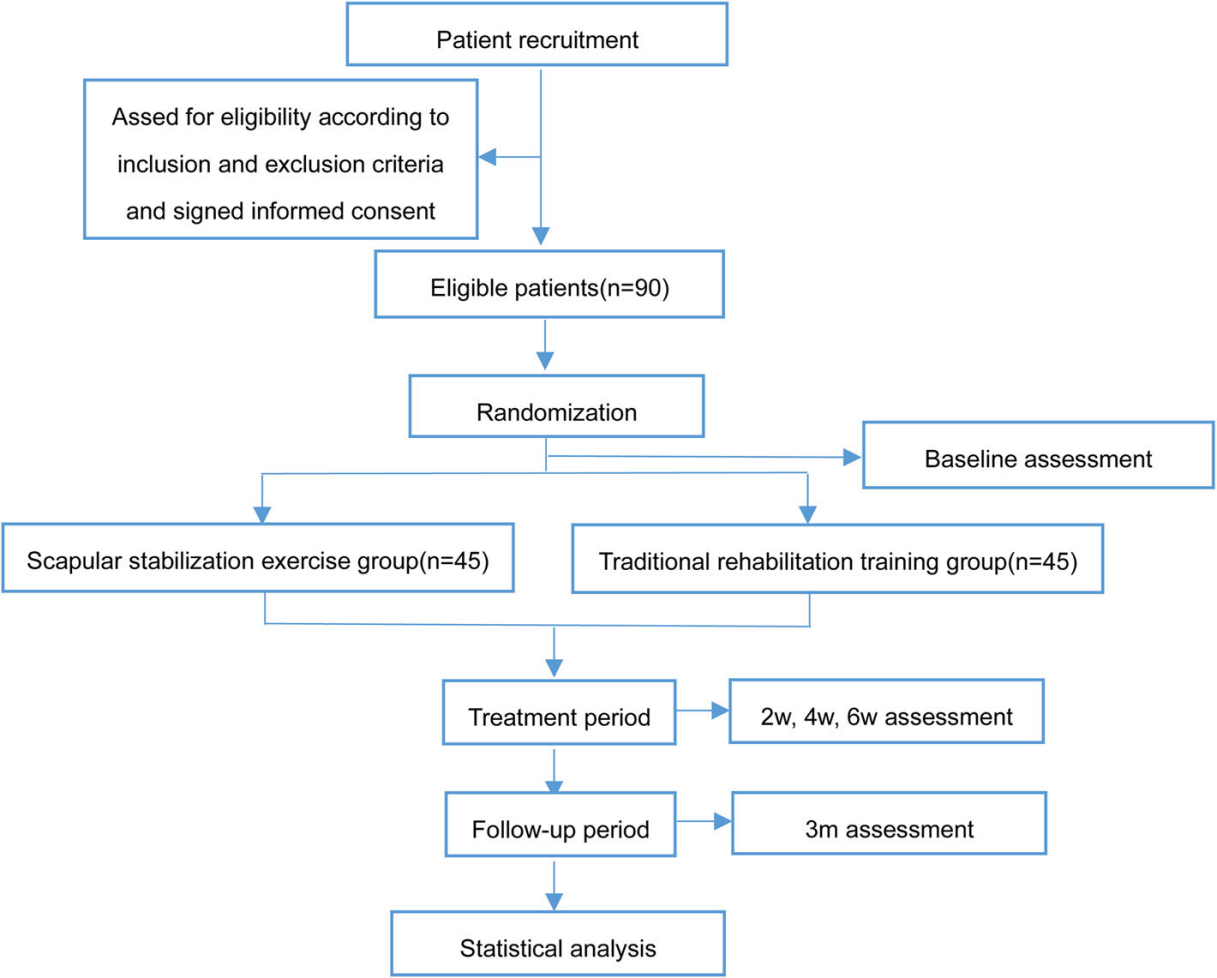
Study flowchart

**Fig. 2:**
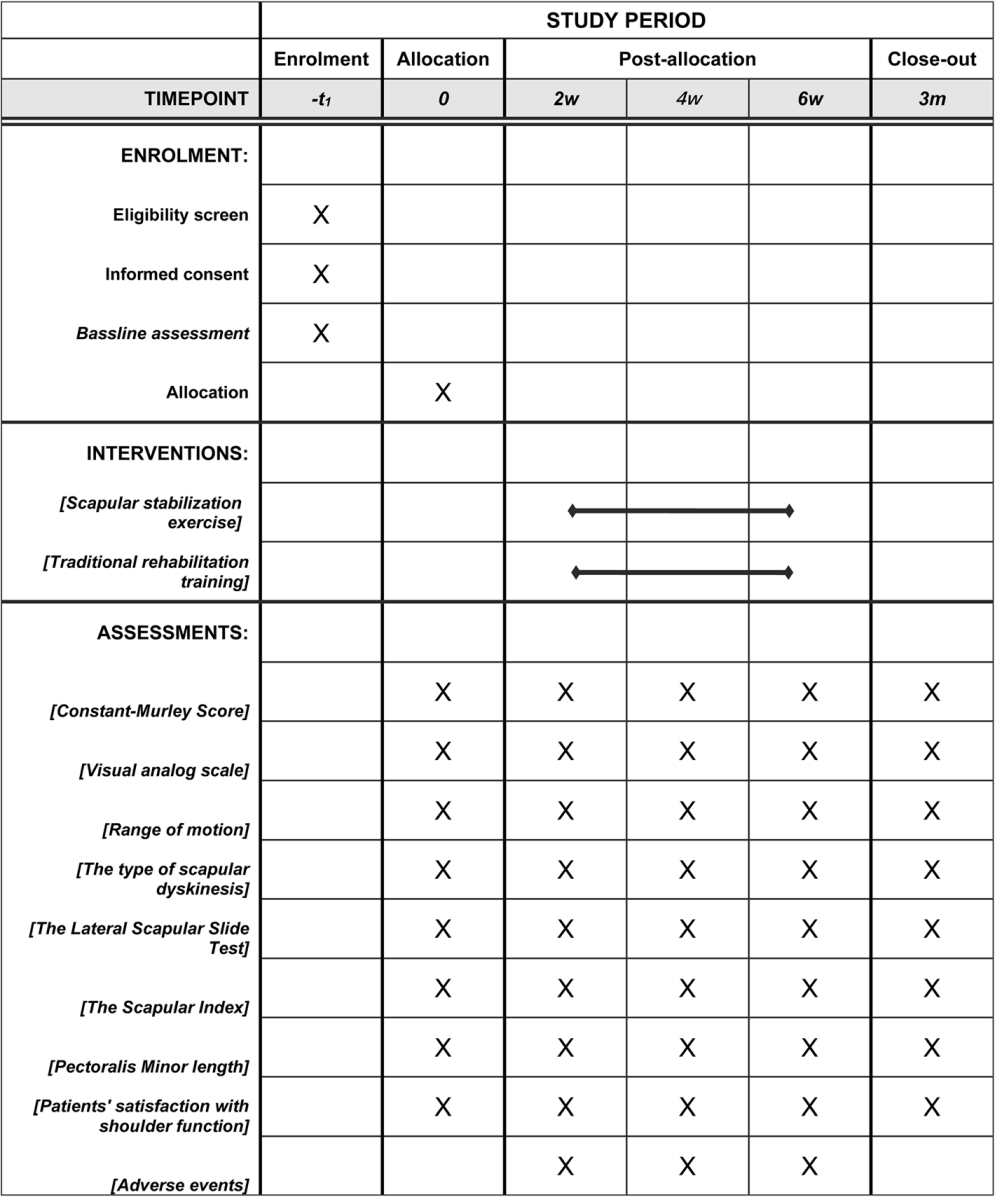
Schedule of enrollment, intervention, study visits, and assessments for both study groups

This is a prospective, multicenter, randomized controlled, parallel-group superiority trial, designed following the SPIRIT 2013 statement. The CONSORT flow chart is presented in Fig. 1. Figure 2 shows the schedule of enrolment, intervention, assessments, and study visits for the study groups. This study will be performed at Shanghai Jiao Tong University Affiliated Sixth People’s Hospital, the Lingang Branch of Shanghai Sixth People’s Hospital, Shanghai 8th People’s Hospital, Shanghai Xuhui District Dahua Hospital, and Shanghai Fengxian District Central Hospital from March 2021 to January 2023. Study participation will be promoted through advertisements. Participants will undergo screenings to confirm that they meet the inclusion criteria and exclusion criteria and will be provided written informed consent to participate. The purpose and content of the study of the study will be clarified by the first researcher to the subjects, and written informed consent will be obtained from them at the time of enrolment. In the consent form, the participants will be asked if they agree to use their data in case they withdraw from the study. Moreover, the research team will get their permission to share relevant data with other academic experts. This experiment does not involve collecting biological specimens. The study was approved by Ethics Committee of Shanghai Sixth People’s Hospital (2021-031) and registered in the Chinese Clinical Trials Registry (ChiCTR2100044332). Evaluators and data collectors will be blinded as to the groups and treatment allocation.

Treatment groups will receive different treatment programs as follows:

- Group 1: Scapular stabilization exercise based on the type of SD for once a day, for up to 6 weeks.
- Group 2: Traditional rehabilitation training for once a day, for up to 6 weeks.

### Inclusion criteria

- Males and females aged 20 to 60 years old.
- Unilateral shoulder pain, with or without limited shoulder movement.
- Patients who meet the diagnostic criteria with broadly defined periarthritis of the shoulder, including rotator cuff injuries, biceps tendonitis, supraspinatus tendonitis, shoulder impingement syndrome, and shoulder instability.
- A maximum pain score of equal or less than 5/10 on a numerical rating scale (NRS)
- Patients volunteered to participate in the study and signed informed consent.

### Exclusion criteria

- Rotator cuff tendon torn in all layers, fracture, dislocation, nerve injury, and other diseases that cause shoulder pain.
- History of trauma or shoulder surgery.
- Patients with scoliosis or acute cervical spondylosis.
- The flexion or abduction angle of the shoulder less than 90°.
- Patients that body surface markers are not obvious, which will affect the assessment, such as obesity (body mass index greater than 28).
- Other diseases that unfit or unable to complete the treatment plan, such as active infections, active inflammatory diseases, tumors, neurological disorders and changes in cognitive function (such as stroke, epilepsy, Parkinson’s disease, and peripheral neuropathy), systemic diseases involving joints (such as rheumatoid arthritis), and severe osteoporosis.
- Patients who refuse to accept the treatment.

### Exit criteria

- Patients who are unable to adhere to the study protocol and study requirements;
- Patients who have severe adverse reactions after intervention and cannot successfully complete the treatment;
- Any new illness that affects their inclusion;
- The patient is lost to follow-up;

### Interventions

Patients recruited to the trail will undergo a 3-day washout period before entering the treatment. If the patient is unable to adhere to the study protocol and study requirements during the washout period, the patient will withdraw from the study. All patients will be treated by the same therapist, who is not blinded to the treatment groups as the therapist will adjust exercise intensity. Patients will exercise at home once a day for 6 weeks and cone for further consultation every 2 weeks. In addition, physical therapy and anti-inflammatory drugs can be appropriately used according to the condition of the patient. To maintain the adherence of the patients to the intervention, the researcher team will regularly contact patients during the intervention via telephone or the WeChat between visits and online clocking software is used to ensure patient compliance.

#### Scapular stabilization exercise based on the type of SD

In this trial, scapular stabilization exercise is designed based on the manifestation of muscle imbalance for each type of SD. For some muscles, there is a consensus that these muscles need to be strengthened such as the MT&LT, SA, and RM. Their activation is important for scapular stabilization because these muscles maintain the optimal scapular position during elevation of the arm: upward rotation, external rotation, and posterior tilt [16]. For other muscles such as the PM, LS, UT, and TM, they need to be inhibited. If PM is too tight, it will lead to an increase in scapular anterior tilting and internal rotation and a decrease in scapular upward rotation, resulting in a reduction of the subacromial space [23]. Therefore, it is necessary to strengthen the weak muscles and stretch the tense ones.

All the scapular stabilization exercises in this trial are selected according to the literature regarding scapular muscle dysfunction and new insights derived from recent research [11, 21, 24–30], combined with muscle anatomy and function and clinical experience. Participants in the scapular stabilization exercise group will follow a supervised 6-week exercise program consisting of strengthening exercises (MT&LT, SA, and RM) and stretching exercises (PM, LS, UT, TM). Appropriate exercises will be given to patients according to the type of SD, and the details of exercises are shown in the Table 2:

- Type-1: PM stretching + SA strengthening + MT&LT strengthening
- Type-2: TM stretching + SA strengthening + MT&LT strengthening + RM strengthening
- Type-3: PM stretching + UT stretching + LS stretching + MT&LT strengthening

**Table 2:** Scapular stabilization exercise

Exercise	Description	Intensity and frequency	Figure
PM stretching	**a.** Subject is standing, the affected arm positioned at wall with elbow flexion into 90°; subject performs stretching with leaning forward. **b.** Subject stands at table and places hands on the edge of the table for support, then performs stretching with squat slowly.	Intensity: It depends on the subjective feeling of the patient—the patient feels slightly tired and slightly shortness of breath (RPE range 11 to 15). Stretching exercise: Each is repeated for 3 times. Strengthening exercise: Each active exercise is progressed from 10 reps*3 sets to 15 reps*3 sets. If subject easily completed 15 reps*3 sets of active exercise, they will go on to resisted exercise. Heavier resistance will be given when the patient can easily complete the lower resistance. Frequency: once a day, for 6 weeks. Time: Stretching exercise: stretch for 5 abdominal breaths at one time, with a 1-min interval between times. Strengthening exercise: the duration of muscle contraction is around 5 seconds, with a 1-min interval between sets.	**a.** **b.**
TM stretching	Subject is standing, slowly performs flexion of the shoulder		
UT stretching	Subject is sitting, with the affected hand pressed under the buttock; subject tips head to the healthy side, and then rotates to the affected side; subject performs stretching with slowly lowering head.		
LS stretching	Subject is sitting, with the affected hand pressed under the buttock; subject tips head to the healthy side, and then rotates to the healthy side; subject performs stretching with slowly lowering head.		
SA strengthening	**Stage 1: active exercise** Subject is standing, with shoulder flexion into 90°; subject slowly performs scapular protraction (A) and retraction (B). **Stage 2: resisted exercise** Subject is standing, holding the elastic band, with shoulder in 90°of forward flexion; subject slowly performs scapular protraction (A) and retraction (B).		**Active exercise** **(A) (B)** **Resisted exercise:** **(A) (B)**
MT&LT strengthening	**Stage 1: active exercise** Exercise starts with the individual in the side-lying position with the shoulder in neutral position and elbow flexed to 90°(A). Perform external rotation of the shoulder with a towel between the elbow and trunk(B). Avoid compensatory movements. **Stage 2: resisted exercise** The dumbbell as resistance for this exercise, other details are the same as the active exercise.		**Active exercise:** **(A)** **(B)** **Resisted exercise:** **(A)** **(B)**
RM strengthening	**Stage 1: active exercise** Sitting, take a deep breath and extend the shoulders back and pull the elbows back, bringing the scapula as close to the spine as possible, hold for 5 seconds, then slowly return to the starting position while exhaling. **Stage 2: resisted exercise** Subject is sitting, both hands holding the elastic band fixed in front of the body, other details are the same as the above active exercise.		**Active exercise:** **Resisted exercise:**

NOTE. “a” means the more difficult exercise for patients with better shoulder function; “b” means the simpler exercise for patients with poor shoulder function. Choose one of the two exercises according to the patient’s actual ability: teach the patient the exercise “b” if he/she is unable to do the exercise “a.”

Resisted exercises are done with elastic bands (Thera-Band) and dumbbells with different resistance.

Abbreviations: *reps* repetitions, *PM* pectoralis minor, *TM* teres major, *LS* levator scapulae, *UT* upper trapezius, *MT&LT* middle&lower trapezius, *SA* serratus anterior, *RPE* rating of perceived exertion

#### Traditional rehabilitation training

In the traditional rehabilitation training group, patients will receive the following traditional rehabilitation exercises for periarthritis of the shoulder: pendulum exercise, wall climbing exercise, external rotation exercise (Table 3).

**Table 3:** Traditional rehabilitation training

Exercise	Description	Intensity and Frequency	Figure
Wall climbing exercise	Slowly climb up the wall with hands, and then slowly return down to the original position.	Intensity: It depends on the subjective feeling of the patient—the patient feels slightly tired and slightly shortness of breath (RPE range 11 to 15). Each exercise starts with 10 reps*3 sets, and increases 5 reps a week. Frequency: once a day, for 6 weeks. Time: The overall exercise time is 20–30 min.	
Pendulum exercise	The healthy arm is placed on the table to support the body, while the affected arm slowly swings back and forth, side to side, and in circles.		
External rotation exercise	Standing or sitting, hold a stick in hands and bend elbows, then push and pull the stick to both sides with the healthy hand, so as to do internal rotate and external rotate.		

### Outcome assessments

All outcome assessments will be evaluated by well-trained personnel who are blind to the treatment allocation. Assessments will be performed at baseline, 2-, 4-, and 6-week treatment, and at the 6-week follow-up after the end of treatment.

#### Primary outcome measures

Constant-Murley Score (CMS) is a widely used scale in the assessment of shoulder function. The successful application of CMS has proven its validity, reliability, and responsiveness for the detection of shoulder pathologies [31]. The minimal clinically important difference (MCID) considered for the score is 17 points [32]. The CMS includes four aspects related to shoulder pathology: pain, activities of daily living (ADL), range of motion (ROM), and muscle strength. Pain and ADL are answered by the patient; ROM and strength are done by doctors [33]. The score ranges from 0 to 100 points, and higher scores mean better function.

#### Secondary outcome measures

The secondary outcome measures include pain degree, ROM, type of SD, scapula position, and patients’ satisfaction with shoulder joint function. The Lateral Scapular Slide Test (LSST), the Scapular Index (SI), and the Pectoralis Minor Index (PMI) are used to assess scapula position.

Pain intensity at rest, during movement, and sleeping at night are assessed using the numerical rating scale (NRS), with 0 being “No pain,” and 10 being “The worst pain” [34]. The NRS has been shown to be a reliable and valid instrument for the assessment of pain intensity [35, 36]. For the ROM assessment, the electronic goniometer is used to measure active flexion and abduction movements and passive flexion and abduction movements. Previous studies have revealed the electronic goniometer have good validity and reliability [37].

The type of SD is assessed by the comprehensive method combined visual observation and palpation [38]. Subjects are evaluated at rest position in a warm and comfortable environment. Raters make the initial assessment by visual observation, and then put hands on the subject’s bilateral scapula for further identification. This method shows satisfactory inter-reliability [35]. The subjects are assessed by three raters with previous experience with assessments of SD, and raters are allowed discussions to derive a unanimous decision. Subjects whose scapular pattern could not be identified by raters or assessment results are inconsistent among raters will be excluded.

LSST is a quantitative method for assessing inferior angle displacement and recognizing scapular symmetry [39]. The distance from the inferior angle of the scapula to the corresponding thoracic spinous process is measured in three positions: the first position is of the arm relaxed at the sides; the second is hands placed on the lateral iliac crest; and the third is 90° of shoulder abduction and in full internal rotation. A difference of more than 1.5 cm between two sides is considered asymmetric.

SI is calculated by measuring the distance from the sternal notch (SN) to the coracoid process (CP) and the horizontal distance from the posterolateral angle of the acromion (PLA) to the corresponding thoracic spine (TS) with a soft tape measure, using the equation: [(SN to CP/PLA to TS) × 100] [40]. The current study supports SI to measure scapular position because it is correlated with scapular internal rotation [37]. An increase in internal rotation at rest should result in a decreased SN to CP distance and an increased TS to PLA distance, which leads to a smaller SI.

Pectoralis minor length (PML) is divided by the participant’s height and multiplied by 100 to calculate PMI [23]. PMI allows measurements to be normalized to participant’s heights to provide resting PML. This method has been proven to have good validity and reliability [41, 42]. PML is defined as the distance between the coracoid process and the inferior medial aspect of the fourth rib adjacent to the sternocostal junction [23]. The assessment is performed using a flexible tape measure, with the participant standing in a relaxed posture with both arms in a neutral position. To avoid the influence of breathing on the measurement results, it is measured immediately after exhalation. It is reported that pectoralis minor tightness can cause increased scapular anterior tilting, internal rotation, and downward rotation [23, 41, 43].

Patients’ satisfaction with shoulder function is also measured with NRS, with 0 meaning “Extremely unsatisfied with shoulder function,” and 10 meaning “Very satisfied with shoulder function.”

### Follow-up

Follow-up will occur 6 weeks after completion of the treatment program. This time point was selected to assess sustained long-term effectiveness of the intervention.

### Sample size calculation

The study is designed to achieve 95% power overall at the 0.05 significance level to detect change on the CMS between groups after 6 weeks. According to G*Power 3.1 software, we use repeated measurement analysis of variance (ANOVA) (Effect size = 0.25, Number of measurements = 4) to calculate the sample size. Considering a dropout rate of 20%, a sample size of 90 (45 per group) will be needed in this trial.

The statistical hypotheses are:

- H0: mean CMS scale (scapular stabilization exercise) = mean CMS scale (traditional rehabilitation training).
- HA: mean CMS scale (scapular stabilization exercise) ≠ mean CMS scale (traditional rehabilitation training).

### Randomization and allocation concealment

Consented eligible participants will be randomly allocated to one of the two groups in a ratio of 1:1. Patients’ allocations will be according to a computer-generated randomization table. Generation of the allocation sequence, enrollment of participants, and assignment of participants to interventions will be done by the main researcher of the trial. When the subject is enrolled into the study, the subject will be assigned a study number sequentially. If the subject drops out of the study, the assigned study number will not be reused. The block size will not be known until the end of the study for allocation concealment. The random numbers will be sealed in envelopes by an individual other than the trial researchers. The envelopes will be randomly selected by the patients, and they would be assigned to the intervention or control groups according to the contents of the envelope.

### Blinding

The therapist and patients will not be blinded because of the nature of the intervention, but it is feasible to make the researchers, outcome evaluators, and statisticians blinded to the group/treatment assignment. Furthermore, the outcome evaluation will be done by well-trained personnel in order to minimize bias. After the completion of data analysis, the statistician will present un-blinded data to the researchers.

### Date management and monitoring

All staff members will be trained to ensure data quality. The researchers will record data in case report forms (CRFs) and any changes to a CRF will be signed and dated. Data administrators will enter data into the computer. Data of the subjects will be stored at the security site. The collected data will be identified by a coded ID number. Data collected during the research will be confidential and only accessed by the researchers. The corresponding author will also have access to the dataset. The original data will be kept in the research center and will be accessible upon reasonable request. Moreover, the anonymous data will be shared with other researchers. The results of the trial will be disseminated through journal publication regardless of the magnitude of the effects.

The Ethics Committee of Shanghai Sixth People’s Hospital will be fully aware of the trials from the first draft of the protocol to the end of the research process and will monitor the accuracy of the trials. The research team will organize a conference to discuss problems encountered and propose solutions every month. The Data Monitoring Committee was not involved in the current trial considering the low-risk intervention.

### Safety monitoring

Patients will be informed of the potential risks and benefits of the trial and sign an informed consent form before participating in the research to protect their rights and interests.

Intervention involved in the study protocol is exercise therapy, so serious adverse events are rare. When potential adverse events occur in a patient, the researchers will record the severity, duration, treatment measures, and event outcomes. The doctor will determine the causal relationship between the treatment and the adverse event and decide whether the patient is suitable for continued participation in the study. Also, relevant information will be reported to the ethics committee.

The following are the criteria for terminating a patient from the study: (1) a serious adverse event happened that the researchers believes that the patient is not suitable for continued participation or the patient has requested to be withdrawn from the study; (2) the patient is found to have serious diseases during the study; (3) the patient has received other treatments that may affect the outcomes.

### Statistical analysis

In this trial, we will implement the intention-to-treat analysis and statistical analysis will be carried out by a researcher not involved in the evaluation and treatment. Data will be recorded in the CRFs. Data analysis will be done using the SPSS version 26.0 statistical software. In order to incorporate the data of the participants into data analysis regardless of the protocol adherence, all the analyses will be performed in accordance with the intention-to-treat principle. Data will be expressed as frequencies and percentages for categorical variables, mean, and (±) standard deviation for continuous variables. Intergroup comparison of the type of SD will be calculated using chi-squared test. Intergroup comparison of pain, ROM, scapula position (LSST, SI, PMI), and patients’ satisfaction with shoulder function will be calculated using repeated measurement ANOVA. Intergroup comparison of time points will be calculated using Least Significant Difference. Statistical significance is defined as a *P* value of < 0.05.

## Discussion

With the change of people’s life style and work style, the incidence of periarthritis of the shoulder is increasing year by year. Early studies have revealed that patients with periarthritis of the shoulder usually have SD, and there is a close relationship between SD and scapular muscle imbalance [11, 18]. Current evidence suggests that scapular control is an important component of scapular rehabilitation and scapular stabilization exercise is effective in reducing pain and improving function [44]. Scapular stabilization exercise that includes stretching and strengthening emphasis scapular position and kinematics, allowing the scapula to perform the role of energy transfer, serving as the basis for muscle activation and as a link in the kinetic chain [45]. However, there is a lack of targeted scapular stabilization exercise based on the type of SD.

To the best of our knowledge, the present study design will be the first randomized clinical trial comparing the efficacy of scapular stabilization exercise based on the type of SD versus traditional rehabilitation training for the treatment of periarthritis of the shoulder. The novelty of this study is maintaining the force couple of scapulae altered due to muscle imbalance and designing targeted exercise based on the type of SD, thereby improving shoulder function, reduce pain and disability for patients with periarthritis of the shoulder. The study will provide data and guidance whether targeted scapular stabilization exercise will show a significant improvement in patients with periarthritis of the shoulder. It is expected that findings from the study will be incorporated into periarthritis of the shoulder treatment guidelines and be translated into future services for patients with periarthritis of the shoulder.

## Trial status

The trial is currently underway. It is planned for the study to complete by January 1, 2023. Any protocol amendments will be updated in ChiCTR.

## Data Availability

The datasets generated and/or analyzed during the current study are not publicly available due to ethical considerations but may be available from the corresponding author on reasonable request.

## Abbreviations

SD: Scapular dyskinesis
UT: Upper trapezius
MT/LT: Middle/lower trapezius
SA: Serratus anterior
PM: Pectoralis minor
TM: Teres major
LS: Levator scapulae
ADL: Activities of daily living
NRS: Numerical rating scale
CMS: Constant-Murley Score
MCID: Minimal clinically important difference
ROM: Range of motion
LSST: The Lateral Scapular Slide Test
SI: The Scapular Index
PMI: The Pectoralis Minor Index
SN: Sternal notch
CP: Coracoid process
PLA: Posterolateral angle of the acromion
TS: Thoracic spine
CRF: Case report form
ANOVA: Analysis of variance
CI: Confidence interval

## References

[1] Wu Z, Yu X, Xiong J, Wu G, Zuo Z, Xie Q (2020). Acupuncture and moxibustion therapy for scapulohumeral periarthritis: protocol for an overview of systematic reviews and meta-analysis. Medicine..

[2] Kothari S, Srikumar V, Singh N (2017). Comparative efficacy of platelet rich plasma injection, corticosteroid injection and ultrasonic therapy in the treatment of periarthritis shoulder. J Clin Diagn Res.

[3] Huang C, Tsao S, Cheng C, Hsin M, Chen C (2010). Treating frozen shoulder with ultrasound-guided pulsed mode radiofrequency lesioning of the suprascapular nerve: two cases. Pain Med.

[4] Favejee M, Huisstede B, Koes B (2011). Frozen shoulder: the effectiveness of conservative and surgical interventions--systematic review. Br J Sports Med.

[5] Lorbach O, Anagnostakos K, Scherf C, Seil R, Kohn D, Pape D (2010). Nonoperative management of adhesive capsulitis of the shoulder: oral cortisone application versus intra-articular cortisone injections. J Shoulder Elb Surg.

[6] Longo U, Risi Ambrogioni L, Berton A, Candela V, Massaroni C, Carnevale A, et al. Scapular dyskinesis: from basic science to ultimate treatment. Int J Environ Res Public Health. 2020;17(8):2974. 10.3390/ijerph17082974. 10.3390/ijerph17082974 PMC7215460 32344746

[7] Depreli Ö, Angın E (2018). Review of scapular movement disorders among office workers having ergonomic risk. J Back Musculoskelet Rehabil.

[8] Kibler W, Uhl T, Maddux J, Brooks P, Zeller B, McMullen J (2002). Qualitative clinical evaluation of scapular dysfunction: a reliability study. J Shoulder Elb Surg.

[9] Kibler W (2012). The scapula in rotator cuff disease. Med Sport Sci.

[10] Kibler W, Sciascia A, Wilkes T (2012). Scapular dyskinesis and its relation to shoulder injury. J Am Acad Orthop Surg.

[11] Huang T, Ou H, Huang C, Lin J (2015). Specific kinematics and associated muscle activation in individuals with scapular dyskinesis. J Shoulder Elb Surg.

[12] Ludewig P, Cook T (2000). Alterations in shoulder kinematics and associated muscle activity in people with symptoms of shoulder impingement. Phys Ther.

[13] Cools A, Witvrouw E, Declercq G, Vanderstraeten G, Cambier D (2004). Evaluation of isokinetic force production and associated muscle activity in the scapular rotators during a protraction-retraction movement in overhead athletes with impingement symptoms. Br J Sports Med.

[14] McClure P, Michener L, Karduna A (2006). Shoulder function and 3-dimensional scapular kinematics in people with and without shoulder impingement syndrome. Phys Ther.

[15] Chester R, Smith T, Hooper L, Dixon J (2010). The impact of subacromial impingement syndrome on muscle activity patterns of the shoulder complex: a systematic review of electromyographic studies. BMC Musculoskelet Disord.

[16] Timmons M, Thigpen C, Seitz A, Karduna A, Arnold B, Michener L (2012). Scapular kinematics and subacromial-impingement syndrome: a meta-analysis. J Sport Rehabil.

[17] Struyf F, Cagnie B, Cools A, Baert I, Brempt J, Struyf P (2014). Scapulothoracic muscle activity and recruitment timing in patients with shoulder impingement symptoms and glenohumeral instability. J Electromyogr Kinesiol.

[18] Castelein B, Cagnie B, Cools A (2017). Scapular muscle dysfunction associated with subacromial pain syndrome. J Hand Ther.

[19] Başkurt Z, Başkurt F, Gelecek N, Özkan M (2011). The effectiveness of scapular stabilization exercise in the patients with subacromial impingement syndrome. J Back Musculoskelet Rehabil.

[20] Struyf F, Nijs J, Mollekens S, Jeurissen I, Truijen S, Mottram S, Meeusen R (2013). Scapular-focused treatment in patients with shoulder impingement syndrome: a randomized clinical trial. Clin Rheumatol.

[21] Turgut E, Duzgun I, Baltaci G (2017). Effects of scapular stabilization exercise training on scapular kinematics, disability, and pain in subacromial impingement: a randomized controlled trial. Arch Phys Med Rehabil.

[22] Hotta G, Santos A, McQuade K, de Oliveira A (2018). Scapular-focused exercise treatment protocol for shoulder impingement symptoms: three-dimensional scapular kinematics analysis. Clin Biomech (Bristol, Avon).

[23] Borstad J, Ludewig P (2005). The effect of long versus short pectoralis minor resting length on scapular kinematics in healthy individuals. J Orthop Sports Phys Ther.

[24] Cools A, Dewitte V, Lanszweert F, Notebaert D, Roets A, Soetens B (2007). Rehabilitation of scapular muscle balance: which exercises to prescribe?. Am J Sports Med.

[25] McClure P, Greenberg E, Kareha S (2012). Evaluation and management of scapular dysfunction. Sports Med Arthrosc Rev.

[26] De Mey K, Danneels L, Cagnie B, Van den Bosch L, Flier J, Cools A (2013). Kinetic chain influences on upper and lower trapezius muscle activation during eight variations of a scapular retraction exercise in overhead athletes. J Sci Med Sport.

[27] Castelein B, Cagnie B, Parlevliet T, Cools A (2016). Serratus anterior or pectoralis minor: which muscle has the upper hand during protraction exercises?. Man Ther.

[28] Camargo P, Neumann D (2019). Kinesiologic considerations for targeting activation of scapulothoracic muscles - part 2: trapezius. Braz J Phys Ther.

[29] Kibler W, Sciascia A (2019). Evaluation and management of scapular dyskinesis in overhead athletes. Curr Rev Musculoskelet Med.

[30] Neumann D, Camargo P (2019). Kinesiologic considerations for targeting activation of scapulothoracic muscles - part 1: serratus anterior. Braz J Phys Ther.

[31] Roy J, MacDermid J, Woodhouse L (2010). A systematic review of the psychometric properties of the Constant-Murley score. J Shoulder Elb Surg.

[32] Henseler J, Kolk A, van der Zwaal P, Nagels J, Vliet Vlieland T, Nelissen R (2015). The minimal detectable change of the Constant score in impingement, full-thickness tears, and massive rotator cuff tears. J Shoulder Elb Surg.

[33] Vrotsou K, Ávila M, Machón M, Mateo-Abad M, Pardo Y, Garin O (2018). Constant-Murley Score: systematic review and standardized evaluation in different shoulder pathologies. Qual Life Res.

[34] Chauny J, Paquet J, Lavigne G, Marquis M, Daoust R (2016). Evaluating acute pain intensity relief: challenges when using an 11-point numerical rating scale. Pain..

[35] Chien C, Bagraith K, Khan A, Deen M, Strong J (2013). Comparative responsiveness of verbal and numerical rating scales to measure pain intensity in patients with chronic pain. J Pain.

[36] Hjermstad M, Fayers P, Haugen D, Caraceni A, Hanks G, Loge J, Fainsinger R, Aass N, Kaasa S, European Palliative Care Research Collaborative (EPCRC) (2011). Studies comparing Numerical Rating Scales, Verbal Rating Scales, and Visual Analogue Scales for assessment of pain intensity in adults: a systematic literature review. J Pain Symptom Manag.

[37] Shamsi M, Mirzaei M, Khabiri S (2019). Universal goniometer and electro-goniometer intra-examiner reliability in measuring the knee range of motion during active knee extension test in patients with chronic low back pain with short hamstring muscle. BMC Sports Sci Med Rehabil.

[38] Huang T, Huang H, Wang T, Tsai Y, Lin J (2015). Comprehensive classification test of scapular dyskinesis: a reliability study. Man Ther.

[39] Shadmehr A, Azarsa M, Jalaie S (2014). Inter- and intrarater reliability of modified lateral scapular slide test in healthy athletic men. Biomed Res Int.

[40] Borstad J (2006). Resting position variables at the shoulder: evidence to support a posture-impairment association. Phys Ther.

[41] Borstad J (2008). Measurement of pectoralis minor muscle length: validation and clinical application. J Orthop Sports Phys Ther.

[42] Rosa D, Borstad J, Pires E, Camargo P (2016). Reliability of measuring pectoralis minor muscle resting length in subjects with and without signs of shoulder impingement. Braz J Phys Ther.

[43] Phadke V, Camargo P, Ludewig P (2009). Scapular and rotator cuff muscle activity during arm elevation: a review of normal function and alterations with shoulder impingement. Rev Bras Fis.

[44] McQuade K, Borstad J, de Oliveira A (2016). Critical and theoretical perspective on scapular stabilization: what does it really mean, and are we on the right track?. Phys Ther.

[45] Takeno K, Glaviano N, Norte G, Ingersoll C (2019). Therapeutic interventions for scapular kinematics and disability in patients with subacromial impingement: a systematic review. J Athl Train.

